# MRI analysis of and factors related to knee injuries in amateur marathon runners

**DOI:** 10.1371/journal.pone.0306257

**Published:** 2024-07-09

**Authors:** Yiying Shen, Wanzhen Yao, Yi Huang, Lingxiao Ye, Jie Liu, Mengxiao Liu, Jianping Ding, Yanjing Zhang

**Affiliations:** 1 Department of Radiology, Hangzhou Cancer Hospital, Hangzhou, Zhejiang, China; 2 Department of Radiology, The Affiliated Hospital of Hangzhou Normal University, Hangzhou, Zhejiang, China; 3 MR scientific Marketing, Diagnostic Imaging, Siemens Healthineers Ltd, Shanghai, China; Hamdard University Hospital, PAKISTAN

## Abstract

**Background:**

Marathons are the most challenging form of running, and amateur athletes may be more prone to injury due to a lack of professional knowledge and instruction in running.

**Purpose:**

To analyze the MRI manifestations of and factors related to knee injuries in amateur marathon runners.

**Subjects:**

Data were collected from a hospital database of 105 qualified amateur marathon athletes (65 males,40 females), between May 2018 and December 2021.

**Field strength/sequence:**

1.5T MR: sagittal fs-PDWI, sagittal T1WI and sagittal 3D-DESS sequence.

**Assessment:**

The MRI manifestations of knee joint injury were analyzed and evaluated by two radiologists.

**Statistical tests:**

The inter-observer agreement on MRI readings was analyzed using the kappa coefficient, and binary logistic regression analysis was employed to identify factors associated with knee injuries.

**Results:**

The overall prevalence of knee cartilage lesions, meniscus lesions and bone marrow edema among amateur marathon runners was 45.7%, 72.4%, and 49.5% respectively. Our analysis revealed that older age (OR = 1.135, P<0.001), higher BMI (OR = 1.236, P = 0.044), and slower pace (OR = 2.305, P = 0.017) were associated with increased risk of articular cartilage disease. Furthermore, older age (OR = 1.425, P<0.001) was identified as a risk factor for meniscal lesions, while older age (OR = 1.088, P = 0.002) was bone marrow edema. Notably, no significant correlation was observed between knee joint injuries of amateur marathon athletes and gender or the monthly running distance (P>0.05).

**Conclusions:**

The occurrence of knee injuries among amateur marathon athletes was highly prevalent, with the patellofemoral joint cartilage and posterior horn of medial meniscus being frequently affected areas. Moreover, age, BMI, running years and pace were significant risk factors of knee joint injury.

## Introduction

The marathon is considered the most challenging form of running, especially for amateur athletes. Van Gent et al. [1] reported that the prevalence of lower limb running injuries ranged from 19.4% to 79.3%, with the knee joint being the most common site of injury. MRI is an important way to diagnose knee injury, and 3D dual-echo steady state (3D-DESS) sequence has demonstrated high sensitivity in detecting cartilage lesions. Horga et al. [2] used 3.0T MRI to find that the subchondral bone marrow edema in the tibia and femoral condyle of the novice runners improved after a marathon race, while injuries to the patellar cartilage, semimembranous tendon, iliotibial band and prepatellar bursa showed more advanced signs. Hoessly et al. [3] and Khan et al. [4] reported that running causes temporary quantitative changes (such as T1ρ and T2 values) in the knee joints of healthy athletes, but does not cause irreversible qualitative harmful effects. Numerous studies have investigated the effects of marathon running on the knee joint using MRI [2–7], yet the findings have been inconsistent. Therefore, T1WI, PDWI and 3D-DESS magnetic resonance imaging techniques were used in this study to summarize the MR Characteristics of knee injury of amateur marathon runners and analyze the risk factors that contribute to knee injuries.

## Materials and methods

### Participants

This study collected relevant data from amateur marathon runners who underwent knee MRI examinations at the Affiliated Hospital of Hangzhou Normal University between May 2018 and December 2021. The inclusion criteria were as follows: (1) running for more than one year with a monthly running distance of over 40km, or participating in at least one official marathon;(2) not professionally engaged in marathon running and no participation in marathon formal training. Exclusion criteria were as follows: (1) poor image quality. (2) cruciate injury. Based on these criteria, a total of 105 amateur marathon athletes were included in the study, comprising 65 males and 40 females, with a median age of 46 (38, 51) years, BMI of 22.1 (20.4, 24) kg/m2, running experience of 3.0 (2.0, 4.5) years, monthly running distance of 135 (97.5, 180) km, and running pace of 6:00 (5:20, 6:30) min/km. The study received ethical approval by the Affiliated Hospital of Hangzhou Normal University Research Ethics Committee and written informed consent was obtained from all participants. The research data were accessed between October 2021 and January 2023.

### Magnetic resonance imaging

All participants underwent knee MRI scans using 1.5T MRI scanners (Magnetom Avanto/ Magnetom Aera, Siemens Healthcare, Germany) with special coil for right knee joint. The subjects took the supine position first. The imaging protocols included sagittal T1weighted imaging (T1WI) sequence in sagittal plane [repetition time (TR), 410 ms; echo time (TE), 11 ms; section thickness, 3 mm; intersection gap, 0.6 mm; field of view (FOV), 16 × 16 cm; back angle, 90°; bandwidth, 140 Hz/Px], fat-suppressed protein density-weighted imaging (fs-PDWI) sequence in the sagittal (TR, 3000 ms; TE, 36 ms; section thickness, 3 mm; intersection gap, 0.6 mm; FOV, 16 × 16 cm; back angle, 140°; bandwidth, 198 Hz/Px) and three-dimensional double-echo steady-state (3D-DESS) sequence in sagittal plane (TR, 21 ms; TE, 8 ms; section thickness, 0.6 mm; intersection gap, 0.12 mm; FOV, 16 × 16 cm; back angle, 25°; bandwidth, 178 Hz/Px). All participants were asked to complete the MRI safety screening form, informed consent form, and questionnaire before undergoing MRI.

### Imaging analyses

The knee cartilage was divided into 6 subregions: patellar cartilage (PC), trochlear cartilage (TC), medial femoral cartilage (MFC), lateral femoral cartilage (LFC), medial tibial cartilage (MTC) and lateral tibial cartilage (LTC). The grading of articular cartilage lesions was evaluated using the modified Outerbridge classification [5].

The knee meniscus was divided into four subregions: anterior horn of medial meniscus (MMA), posterior horn of medial meniscus (MMP), anterior horn of lateral meniscus (LMA) and posterior horn of lateral meniscus (LMP). Stoller scale [8] was used to evaluate the classification of meniscus lesions, in which grade 1 and 2 represented meniscus degeneration, and grade 3 represented meniscus tear.

Bone marrow edema (BME) was defined as the area with increased signal intensity on PDWI and decreased signal intensity on T1WI. The WORMS scoring criteria were used to grade BME [9].

Two specialized musculoskeletal radiologists independently diagnosed and recorded the MR Manifestations of knee cartilage, meniscus and bone marrow. In case where there were discrepancies between the radiologists’ reports, a consensus was reached through discussion.

### Statistical analyses

Statistical analysis was performed with SPSS Version 26.0 software. We compared the inter-observer agreement on the MRI readings with kappa coefficient analysis, which takes into account the degree of disagreement between radiologists. Kolmogorov-Smirnov test measurement data do not conform to normal distribution, measurement data is expressed by M (Q1, Q3); Counting data is expressed as a percentage (%).

Multivariate binary logistic regression was used to analyze all relevant factors (gender, age, BMI, running age, the monthly running distance, pace) that may potentially affect knee joint injury. Specific methods: first conduct single-factor binary logistic regression analysis, screen out all the influencing factors that meet the requirements of P<0.1, and finally include them into the multi-factor binary logistic regression analysis. If P<0.05, it will be recognized as statistically significant. The results of the logistic regression analysis were expressed as odds ratios (OR) with corresponding 95% confidence intervals [95% confidence intervals (CI)].

## Results

**Fig 1:**
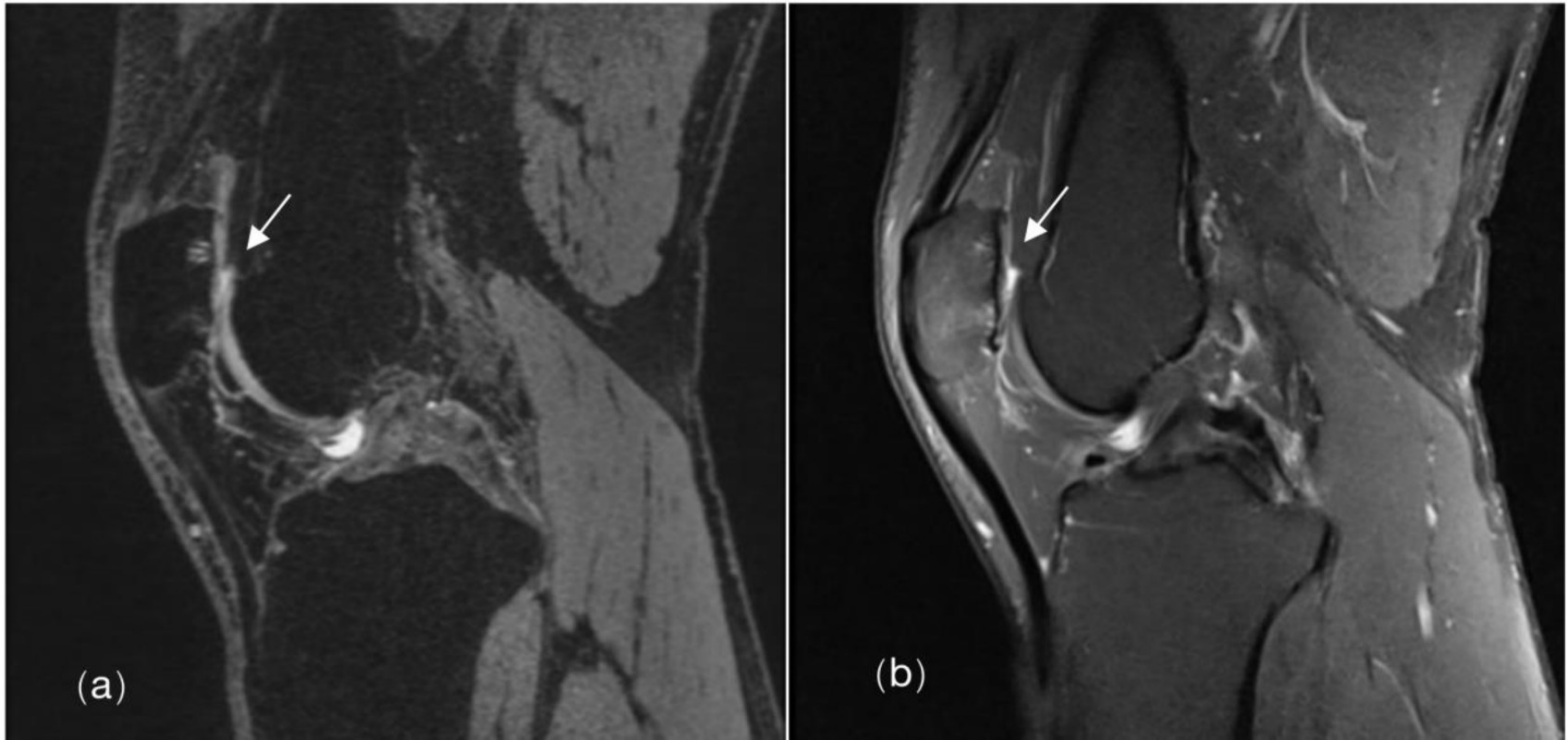
Cartilage lesion. Male amateur marathon runner, 41 years old, BMI 26.1kg/m^2^, 7 running years, monthly running distance 45km, pace 5:00min:sec/km. 3D-DESS (a) and sagittal fs-PDWI (b) showed full-layer defect of right patellar cartilage, exposure of subchondral bone (grade 4) (↑).

**Fig 2:**
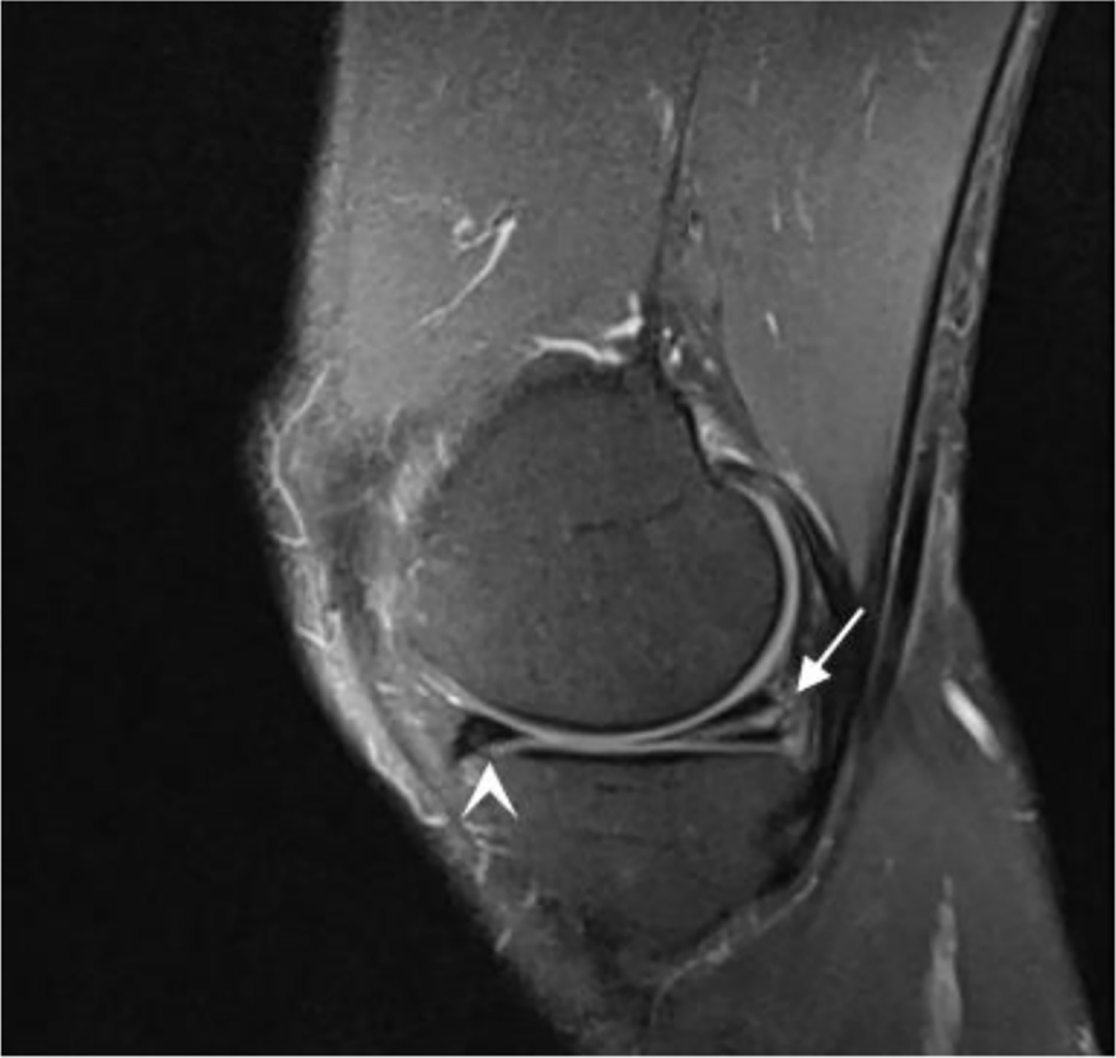
Meniscus lesion. Male amateur marathon runner, 50 years old, BMI 22.2kg/m^2^, running years 4.5 years, monthly running distance 203km, pace 7:00min:sec/km. Sagittal fs-PDWI shows a linear high-signal shadow in the posterior corner of the medial meniscus, involving the articular surface (grade 3) (↑), and a laminate high-signal shadow in the anterior corner of the medial meniscus, reaching the edge of the articular capsule but not reaching the articular surface of the meniscus (grade 2) (arrow).

**Fig 3:**
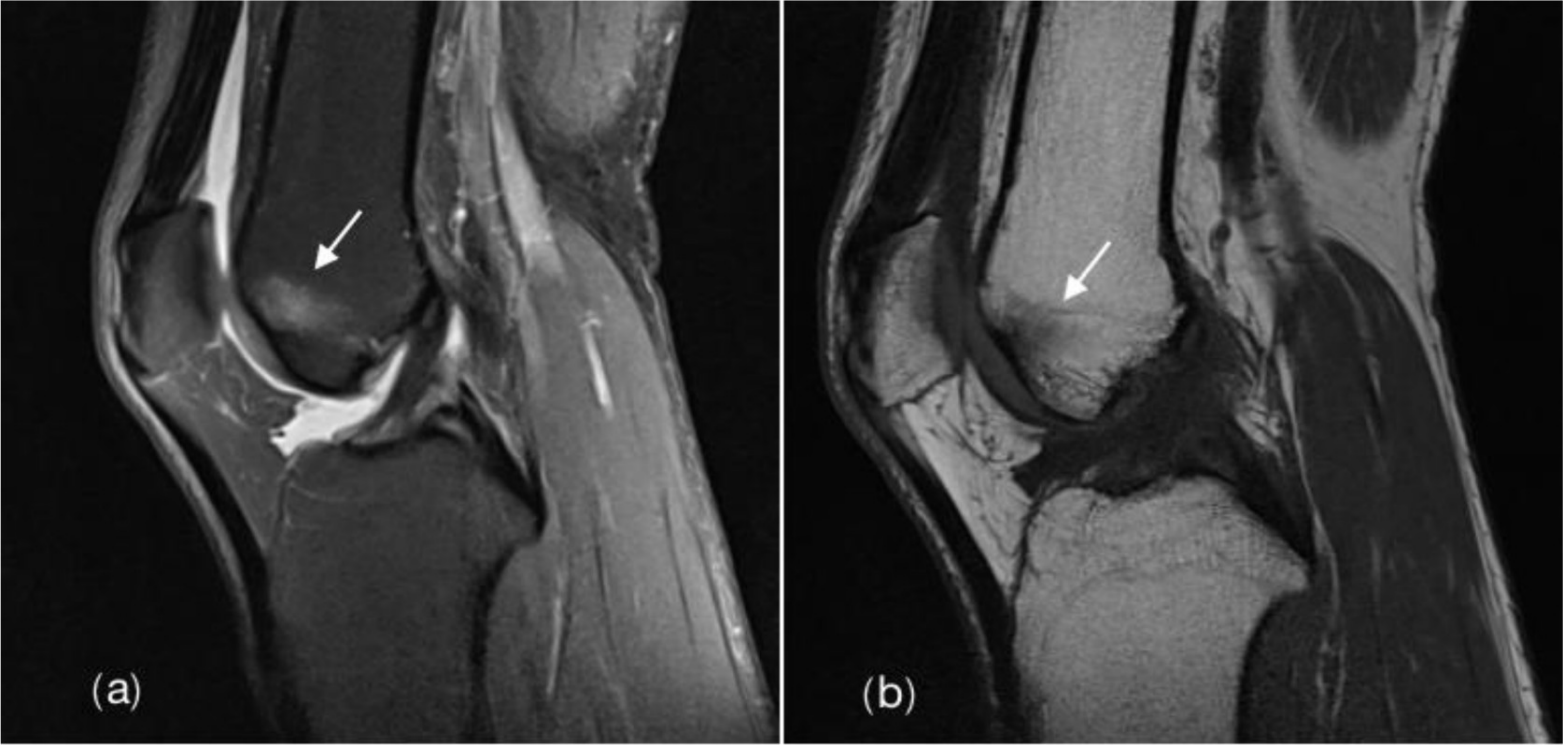
Bone marrow edema. Male amateur marathon runner, 53 years old, BMI22.8kg/m^2^, 1 running year., monthly running distance 90km, pace of 6:00min:sec/km. Sagittal fs-PDWI (a) showed patchy high-signal shadow inside the lateral condyle of the right femur (↑), sagittal T1WI (b) showed patchy low-signal shadow inside the lateral condyle of the right femur (↑), with blurred boundary (grade 2).

The total prevalence of knee cartilage lesion (Fig 1), meniscus lesion (Fig 2) and bone marrow edema (Fig 3) among amateur marathon runners were 45.7% (48/105 subjects), 72.4% (76/105 subjects) and 49.5% (52/105 subjects), respectively. The majority of these lesions were classified as grade 1 and 2 changes (Table 1).

**Table 1:** Classification of knee joint injury [example (%)].

	Total injuries	Grade 0	Grade 1	Grade 2	Grade 3	Grade 4	Inter-observer kappa
**Knee cartilage**	48(45.7)	57(54.3)	14(13.3)	23(21.9)	5(4.8)	6(5.7)	0.818
**Meniscus**	76(72.4)	29(27.6)	34(32.4)	40(38.1)	2(1.9)	/	0.915
**Bone marrow edema**	52(49.5)	53(50.5)	38(36.2)	10(9.5)	4(3.8)	/	0.860

Note: The area with the most serious degree of knee cartilage, meniscus and bone marrow edema was defined as the lesion degree of knee cartilage, meniscus and bone marrow edema.

Among the knee joint cartilage lesions, 31 were classified as grade 1, 39 as grade 2, 5 as grade 3, and 6 as grade 4. The most frequently affected areas were the patellar cartilage (32/105, 30.5%), trochlear cartilage (27/105, 25.7%), and medial femoral cartilage (10/105, 9.5%) (Table 2).

**Table 2:** Regional distribution of knee cartilage [example (%)].

Location	Total injuries	Grade 0	Grade 1	Grade 2	Grade 3	Grade 4	Inter-observer kappa
**PC**	32(30.5)	73(69.5)	19(18.1)	8(7.6)	2(1.9)	3(2.9)	0.897
**TC**	27(25.7)	78(74.3)	7(6.7)	14(13.3)	3(2.9)	3(2.9)	0.76
**MFC**	10(9.5)	95(90.5)	0(0)	10(9.5)	0(0)	0(0)	0.819
**LFC**	7(6.7)	98(93.3)	1(1.0)	6(5.7)	0(0)	0(0)	0.924
**MTC**	2(1.9)	103(98.1)	1(1.0)	1(1.0)	0(0)	0(0)	0.747
**LTC**	3(2.9)	102(97.1)	3(2.9)	0(0)	0(0)	0(0)	0.795

Regarding meniscal lesions, a total of 172 grade 1 lesions, 77 grade 2 lesions, and 4 grade 3 lesions were observed in the amateur marathon athletes. The prevalence of meniscal lesions in each region was posterior horn of the medial meniscus (73/105,69.5%), posterior horn of the lateral meniscus (64/105,61.0%), anterior horn of the lateral meniscus (59/105,56.2%), and anterior horn of the medial meniscus (57/105,54.3%) (Table 3).

**Table 3:** Regional distribution of knee meniscus [example (%)].

Location	Total injuries	Grade 0	Grade 1	Grade 2	Grade 3	Inter-observer kappa
MMA	57(54.3)	48(45.7)	43(41.0)	14(13.3)	0(0)	0.937
MMP	73(69.5)	32(30.5)	34(32.4)	37(35.2)	2(1.9)	0.873
LMA	59(56.2)	46(43.8)	46(43.8)	12(11.4)	1(1.0)	0.843
LMP	64(61.0)	41(39.0)	49(46.7)	14(13.3)	1(1.0)	0.832

The results showed that older age (OR = 1.135, P<0.001), higher BMI (OR = 1.236, P = 0.044), and slow pace (OR = 2.305, P = 0.017) were associated with an increased risk of articular cartilage disease. Older age (OR = 1.425, P<0.001) was also found to be associated with a higher risk of meniscal lesions. Furthermore, older age (OR = 1.088, P = 0.002) and longer running experience (OR = 1.370, P = 0.018) were identified as risk factors for bone marrow edema. However, no significant correlation was found between gender, monthly running distance and knee injury (P>0.05) (Table 4).

**Table 4:** Summary of related analysis results.

MRI features and related factors	Univariate analysis		Multivariate analysis	
	P	OR (95%CI)	P	OR (95%CI)
**Knee cartilage lesions**				
Sex	0.136	1.833(0.827~4.066)		
Age	**0.000**	**1.156(1.084~1.232)**	**0.000**	**1.135(1.059~1.217)**
BMI	**0.008**	**1.262(1.062~1.499)**	**0.044**	**1.236(1.006~1.520)**
Running years	0.772	0.972(0.803~1.177)		
Monthly running distance	0.176	0.996(0.990~1.002)		
Running pace	**0.000**	**2.990(1.638~5.457)**	**0.017**	**2.305(1.162~4.573)**
**Meniscus disease**				
Sex	0.638	1.239(0.507~3.027)		
Age	**0.000**	**1.351(1.201~1.521)**	**0.000**	**1.425(1.225~1.659)**
BMI	**0.045**	**1.222(1.005~1.487)**	0.174	1.194(0.924~1.543)
Running years	0.544	1.075(0.851~1.357)		
Monthly running distance	0.104	1.006(0.999~1.013)		
Running pace	**0.094**	**1.628(0.920~2.878)**	0.078	0.473(0.205~1.088)
**Bone marrow edema**				
Sex	0.128	1.862(0.837~4.142)		
Age	**0.002**	**1.088(1.032~1.146)**	**0.002**	**1.088(1.032~1.146)**
BMI	0.634	1.039(0.888~1.215)		
Running years	0.698	1.038(0.858~1.256)		
Monthly running distance	0.454	1.002(0.997~1.008)		
Running pace	0.250	1.343(0.813~2.218)		

## Discussion

### 1. Analysis of knee cartilage lesions and influencing factors in amateur marathon runners

The study revealed a total prevalence of knee cartilage lesions in amateur marathon runners of 42.3%, primarily falling into grade 1 and grade 2 categories. This result aligns with previous studies reporting a prevalence ranging from 18% to 65% in marathon runners [2, 5]. In addition, it is worth noting that the majority of cartilage lesions in this study were distributed in the patellofemoral joint (73%), having consistence with the results of Horga et al.’s study [2]. Moreover, quantitative MRI studies by Wang et al. [10] and Chen et al. [11] also found that T1ρ and T2 values of patellofemoral cartilage had the most significant changes after running compared with other knee structures. One possible reason is that the patella frequently moves in the trochlear groove during running, and the patellar cartilage is in close contact with the trochlear cartilage [11]. In addition, patellofemoral joints bear more load than tibiofemoral joints in activities involving high knee flexion [12]. Therefore, we believe that the patellofemoral joint may bear more stress load during running, and the high load of the patellofemoral joint may cause runners to develop patellofemoral pain syndrome [13].

The results of this study revealed that age and higher BMI were influencing factors for knee cartilage lesions in amateur marathon runners. The risk of knee cartilage lesions increased with age (OR = 1.135), potentially due to the accumulation of advanced glycation end products and collagen crosslinking. With the increase of age, the accumulation of advanced glycosylation end products and the cross-linking of collagen fibril lead to the increase of the stiffness of collagen matrix, which makes the fibril more prone to fatigue failure, limiting the adaptability of articular cartilage to compression load [14]. Furthermore, the study found that a higher BMI (OR = 1.236, P = 0.044) was associated with an increased risk of knee cartilage lesions in amateur marathon runners. This finding is consistent with a study by Wang et al. [10], which reported a higher risk of cartilage degeneration in runners with a higher BMI. The increased joint load resulting from higher body weight can exceed the physiological mechanical load limits, leading to decreased synthesis of extracellular matrix components and increased activity of pro-inflammatory cytokines. These changes can induce the catabolic metabolism of chondrocytes, potentially contributing to the development of osteoarthritis [15]. Therefore, Gessel et al. [16] suggested that obese individuals should focus on walking to lose weight, gradually incorporating a combination of running and walking, and eventually adapting to continuous running.

In addition, this study showed that slow pace (OR = 2.305, P = 0.017) was associated with increased risk of articular cartilage disease among amateur marathon runners. One possible explanation for this finding is that the cumulative load on the knee joints tends to increase during deceleration, which can contribute to a higher risk of cartilage lesions.

A study by Petersen et al. [17] supports this notion by demonstrating that increasing running speed from 8 km/h to 12 km/h, while maintaining the same distance, can reduce the cumulative impulse on the knee joint by 27%. Therefore, it suggests that higher running speeds may help alleviate the load on the knee joints, potentially reducing the risk of cartilage damage. Additionally, the study suggests that slow walking speed can increase contact and stress on the patellofemoral joint, making runners more susceptible to pre-knee pain [18]. This finding underscores the importance of maintaining an appropriate pace during running activities to minimize the strain on the knee joints and reduce the likelihood of developing articular cartilage disease.

### 2. Analysis of knee meniscus lesions and influencing factors in amateur marathon runners

The study revealed a total prevalence of 72.4% for meniscus lesions among amateur marathon runners, with grade 1 and grade 2 degeneration being the most common manifestations, and only four cases exhibiting grade 3 meniscus tears. Beals et al. [6] analyzed 7 studies on the prevalence of meniscus lesions in asymptomatic runners, and found that 54.5% of meniscus’ signal changes suggested mucoid degeneration, and 5.4% of meniscus tears. The prevalence of meniscus in this study was slightly higher than that reported in the literature, possibly due to the older age of our subjects (median: 46 years, range: 24 to 58 years). In addition, in this study, the prevalence of posterior Angle of medial meniscus was the highest (69.5%), which was consistent with the findings of Horga et al. [2] and Zarins et al. [19]. This may be related to the anatomical structure and biomechanical properties of meniscus.

First, the medial meniscus is characterized by low motion, large diameter and thin thickness [19]. Secondly, during knee flexion, the lateral condyle of femur is higher than the medial condyle, creating a varus force that leads to increased pressure on the medial tibiofemoral joint [11]. Moreover, the medial tibiofemoral joint typically bears 60% to 80% of the knee joint’s pressure load [19]. Furthermore, there exists a posterior inclination between the articular surface of the tibial plateau and the longitudinal axis of the tibial shaft. When subjected to axial loads, the femoral condyle shifts backward and downward, resulting in greater load on the posterior tibiofemoral joint [20]. Therefore, the posterior angle of the medial meniscus endures more stress during running and is more susceptible to damage.

In this study, older age (OR = 1.425, P<0.001) was associated with increased risk of meniscal lesions. Notably, Shellock et al. [21] reported that the prevalence of meniscus tears among marathon runners was not significantly higher than that among sedentary runners, and the extent of meniscal degradation in runners resembled that observed in non-runners. Furthermore, Englund et al. [22] found that meniscus injuries ware notably prevalent in the general population and tended to increase with age. Consequently, it is possible that the meniscus lesions observed in amateur marathon runners might be attributed to age-related degeneration rather than acute injury.

### 3. Analysis of knee bone marrow edema and its influencing factors in amateur marathon runners

The findings from this study revealed that among amateur marathon runners, the prevalence of knee bone marrow edema was 49.5%, primarily characterized by grade 1 and grade 2 changes. Some studies [5, 7, 9] have reported a range of bone marrow edema prevalence among long-distance runners, spanning from 10% to 62.5%.

This study also established a link between older age (OR = 1.088, P = 0.002) and an elevated risk of bone marrow edema. In contrast, Mandalia et al. [23] observed that the prevalence of bone marrow edema in 50 asymptomatic high-level college athletes correlated with prolonged seasonal training, with no significant association identified between bone marrow edema and age or years of exercise. This difference could stem from the fact that our study encompassed older participants (median age: 46 years, range: 24 to 58 years) with lower training intensity compared to high-level athletes. Conversely, Mandalia et al.’s study exclusively involved college students (aged 19 to 23 years) at a higher level of competition.

Moreover, our study did not identify any statistically significant correlation between knee injuries among amateur marathon runners and factors such as gender or the monthly running distance (P > 0.05). This aligns with the findings of a systematic review conducted by Hollander et al. [24], which demonstrated no discernible difference in the overall injury rates between female and male runners. A meta-analysis by Coburn et al. [13] similarly reported that male and female cartilage exhibited similar responses to a single run, with gender showing no influence on changes in cartilage morphology or composition. It has been reported that running more than 92km per week increases the risk of knee injury [25], while only one amateur marathon runner in this study ran more than this distance. Furthermore, the sample size needs to be expanded in the future to verify the association with gender or the monthly running distance and knee injury.

## Limitations

There are limitations in our study. (1) All the subjects in our study were amateur marathon runners who did not receive arthroscopy to verify the diagnostic results of MRI. However, the diagnostic performance of MRI in detecting abnormal knee joints has been verified in a large number of literatures. (2) No sedentary people was recruited as control group to determine whether the knee lesions were caused by running or aging; (3) The knee joint of amateur marathon runners before and after exercise was not compared and dynamically tracked; (4) The included potential influencing factors were few, and possible running-related factors such as the number of marathon races, previous injuries, weekly running times, weekly running time, foot strike pattern, footwear, and running ground conditions were not analyzed.

## Supporting information

S1 File Data. (XLSX)
